# Determinants of good vitamin A consumption in the 12 East Africa Countries using recent Demographic and health survey

**DOI:** 10.1371/journal.pone.0281681

**Published:** 2023-02-16

**Authors:** Maereg Wolde, Zemenu Tadesse Tessema

**Affiliations:** 1 Department of Health Education and Behavioral Sciences, Institute of Public Health, College of Medicine and Health Science, University of Gondar, Gondar, Ethiopia; 2 Department of Epidemiology and Biostatics, Institute of Public Health, College of Medicine and Health Science, University of Gondar, Gondar, Ethiopia; 3 Department of Epidemiology and Preventive Medicine, School of Public Health and Preventive Medicine, Monash University, Melbourne, Victoria, Australia; Università degli Studi di Milano, ITALY

## Abstract

**Background:**

Vitamin A one of the important micronutrients that it cannot be made in the human body and must be taken from outside the body through the diet. Ensuring that vitamin A is available in any form in sufficient quantities remains a challenge, especially in regions where access to vitamin A-containing foods and healthcare interventions is limited. As a result, vitamin A deficiency (VAD) becomes a common form of micronutrient deficiency. To the best of our knowledge, there is limited evidence on determinants of good Vitamin A consumption in East African countries. Therefore, this study aimed to assess the magnitude and determinants of good vitamin A consumption in East African countries.

**Methods:**

A recent Demographic and Health Survey (DHS) of twelve East African countries were included to determine the magnitude and determinants of good vitamin A consumption. A total of 32,275 study participants were included in this study. A multilevel logistic regression model was used to estimate the association between the likelihood of good vitamin A-rich food consumption. Both community and individual levels were used as independent variables. Adjusted odds ratio and its 95% confidence interval were used to see the strength of the association.

**Result:**

The pooled magnitude of good vitamin A consumption was 62.91% with a 95% CI of 62.3 to 63.43. The higher proportion of good vitamin A consumption 80.84% was recorded in Burundi and the smallest good vitamin A consumption 34.12% was recorded in Kenya. From the multilevel logistic regression model, women’s age, marital status, maternal education, wealth index, maternal occupation, children’s age in a month, media exposure, literacy rate, and parity were significantly associated with good vitamin A consumption in East Africa.

**Conclusion:**

The magnitude of good vitamin A consumption in twelve East African countries is low. To increase good vitamin A consumption health education through the mass media and enhancing the economic status of women is recommended. Planners and implementers should give attention and priority to identified determinants to enhance good vitamin A consumption.

## Introduction

Vitamin A, which prevents blindness and infection, has special importance for children [1]. Vitamin A is important in that it cannot be made in the human body and must be taken from outside the body through the diet [2]. Ensuring that vitamin A is available in any form in sufficient quantities remains a challenge, especially in regions where access to vitamin A-containing foods and healthcare interventions is limited [3]. As a result, vitamin A deficiency (VAD) becomes a common form of micronutrient deficiency [4].

Good vitamin A consumption refers to the intake of foods rich in vitamin A at least one food item among the main type of food items while VAD is the result of two primary factors, high frequency of infections, and poverty-related problems [5]. Moreover, socio-cultural limitations and poor absorption leading to impoverished vitamin A stores in the body have been regarded as potential determinants of the prevalence of VAD [6].

A deficiency of vitamin A in the mother leads to low levels of vitamin A in breast milk, inadequate dietary intake of vitamin A during and after weaning, and prevalent illness causes of vitamin A deficiency in young children [7]. The quality and variety of foods reflect the adequacy of vitamin A. The quality of the diet and vitamin A levels are declining in mothers and young children. This is likely the result of changing diets to include foods of animal origin with lower levels of vitamin A and, to a lesser extent, vegetables and fruits [8].

Children and their mothers are most susceptible to its consequences, which include a range of VADD [7,9]. Worldwide, it is estimated that more than 124 million children require vitamin A [10]. Children require extra vitamin A because they are not consuming enough in their normal diet. Whereas, food items with inadequate vitamin A lead to depletion of serum vitamin A levels which affects tissue development, metabolism, and resistance to infections [6]. Also, lack of vitamin A is a risk factor for blindness and mortality from measles and diarrhea in children aged 6 to 59 months [5,10–13]. One study found that a significantly greater proportion of subjects who had VAD were hospitalized, compared to those who were not deficient. The only person who died was with impaired vitamin A [14].

A deficiency can be effectively prevented in a complementary manner through approaches that include supplementation, fortification, biofortification, and improving dietary diversity, with the choice of approaches guided by urgency, epidemiological patterns, and local resources [6,9,13,15].

The prevalence of VAD has declined worldwide, possibly due to widespread vitamin A supplementation in combination with measles vaccination in at-risk populations [3]. While Vitamin A deficiency affects the poorest segments of the population, particularly those in low- and middle-income countries [9,13,16–18]. Therefore, in low- and middle-income countries, routine vitamin A supplementation (VAS) is a key strategy to reduce vitamin A deficiency, mortality, and morbidity in preschool children [19]. VAS is an effective strategy for preventing vitamin A deficiency disorders (VADD) but does not sustainably improve the vitamin A status of a population, does not address underlying causes, and does not reach women of reproductive age [9].

Estimates indicate that the new supplementation regimen will permit a typical child in a developing country setting to attain minimally adequate vitamin A stores during the first 2 years of life [7]. Furthermore, Improved vitamin A nurture alone could prevent 1.3–2.5 million of the nearly 8 million late infancy and preschool-age child deaths that occur each year in the highest-risk developing countries [20]. Effective and long-term control of vitamin A deficiency may be among the most cost-effective and effective interventions for child survival [12].

Controlling vitamin A deficiency is a public health imperative [21]. To deal with vitamin A insufficiency, policymakers view dietary modification that leads to the consumption of vitamin A-rich foods as the safest and most appropriate long-term measure to control vitamin A deficiency [22].

However, some cultural beliefs limit good consumption of vitamin A-rich foods, and adequate consumption of vitamin A-rich foods is also compromised by the cost of these foods, as well as by local cooking, storage, preservation techniques, and seasonality of these foods [23]. The availability of foods with high levels of vitamin A is often seasonal. For example, in some regions, the rainy season is marked by abundant wild leafy vegetables. When liver retinol reserves are low or vitamin A status is compromised by illness, seasonal fluctuations may cause periods of higher risk of deficiency of vitamin A [24].

VAD is a major global public health problem that makes a significant contribution to the global burden of disease [3]. Moreover, it remains a major public health issue in underserved and undernourished areas of the world [9].

Vitamin A deficiency is very common in the African continent. Vitamin A deficiency affects more than 30 million children, most of whom are younger than five years of age [25]. Among the many challenges Africa will have to face in the next few years, vitamin A deficiency can be overcome considering that the need is urgent and that solutions are known, effective, and affordable [12]. In 2013, the prevalence of deficiency was highest in sub-Saharan Africa [10].

In many Eastern and Southern African countries, between one-third and half of the children suffer from VAD [15]. VAD is a major nutritional public health problem in children under the age of five in developing countries, including Ethiopia, Kenya, Nigeria, and South Africa [26,27]. Whereas, VAD is a serious health problem in East Africa. The main reason for the high rate of vitamin A deficiency is an inadequate diet. Much of the population does not consume enough food to meet its calorie requirements, and the variety and nutritional quality of the food are often poor. In addition, the high frequency of infectious diseases raises the risk of vitamin A deficiency [15]. While 62% of children aged 6–23 months had poor vitamin A food intake in Ethiopia [17,28,29].

Even though good Vitamin A consumption has a significant contribution to the reduction of morbidity and mortality of infants and children, as far as our knowledge is concerned, there is no study regarding the magnitude and determinants of good Vitamin A consumption in East African countries. Thus, this study is conducted to determine the magnitude and determinants of good Vitamin A consumption in twelve East African countries.

## Methods

### Data source, tool, and sampling procedure

The data was obtained from the measure DHS program at www.measuredhs.com after preparing a concept note about the project. The DHS program collects data across over 90 low- and middle-income countries across the world. The collected data is comparable for each country. The program implemented the same variable code, variable name, manual, data collection tool, and sampling procedure. Therefore, this study was performed according to relevant DHS statistics guidelines [30]. The Demographic and Health Survey (DHS) data were pooled from the 12 East Africa Countries from 2008 to 2017. The recent DHS of Country-specific datasets was extracted during the specified time. The 12 East Africa Countries from which data were extracted include Burundi, Ethiopia, Kenya, Comoros, Madagascar, Malawi, Mozambique, Rwanda, Tanzania, Uganda, Zambia, and Zimbabwe. The DHS program adopts standardized methods involving uniform questionnaires, manuals, and field procedures to gather information that is comparable across countries in the world. DHSs are nationally representative household surveys that provide data from a wide range of monitoring and impact evaluation indicators in the area of population, health, and nutrition with face-to-face interviews of women aged 15 to 49. The surveys employ a stratified, multi-stage, random sampling design. Information was obtained from eligible women aged 15 to 49 years in each country. **“**The DHS program surveyed according to following sampling procedures. The surveys employ a stratified, multi-stage, random sampling design. First stage: Enumeration Areas (EA) are generally drawn from each country’s Census files. Second stage: in each EA selected, a sample of households is drawn from an updated list of households. Information was obtained from eligible women aged 15 to 49 years in each country. Detailed survey methodology and sampling methods used in gathering the data have been reported elsewhere [31].

### Sample size

A total of 32,275 study participants were included in this study. This include Burundi (4,323), Ethiopia(3,240),Kenya(5,576),Comoros(870),Madagascar(1,725),Malawi(1,641),Mozambique(3,500),Tanzania(1,195),Rwanda(3,082),Uganda(1,429),Zambia(3,811),and Zimbabwe(1,877). Missing data were excluded from the analysis.

### Variables

#### Outcome variable

Living children aged 6–23 months living with their mother who consumed foods rich in vitamin A at least one food item among the seven food items (1) Have the child taken eggs in the last 24 hours?2) Have the child taken meat (beef, pork, lamb, chicken, etc.) in the last 24 hours? 3) Have the child taken a pumpkin, carrots, and squash (yellow or orange inside) in the last 24 hours? 4) Have the child taken any dark green leafy vegetables in the last 24 hours? 5) Have the child taken mangoes, papayas, and another vitamin A fruit in the last 24 hours? 6) Have the child was taken liver, heart, and other organs in the last 24 hours? 7) Have the child taken fish or shellfish in the last 24 hours?) at any time in the last 24 hours preceding the interview was declared good consumption of foods rich in vitamin A coded as “1”, whereas, no consumption of foods rich in vitamin A in the 24 hours preceding the interview was poor consumption coded as “0”.

#### Independent variables

Based on known facts and literature the independent variables included in this was two types of variable that are individual-level and community-level variables. Community-level variables Country and residence. The individual-level variables are Age group, marital status, Educational status, literacy level, Occupational status, parity, children’s age in a month, family size and wealth index, breastfeeding status, and media exposure.

### Data management and analysis

The data was cleaned by STATA version 14.1 software. Sample weighting was done for further analysis.

### Multi-level analysis

Since the outcome variable was binary two-level mixed-effects logistic regression analysis was employed. Sampling weight was applied as part of a complex survey design using the primary sampling unit, strata, and women’s weight (V005).

The individual and community-level variables associated with good vitamin A consumption were checked independently in the bivariable multilevel logistic regression model and Variables that were statistically significant at a p-value of 0.2 in the bivariable multilevel mixed-effects logistic regression analysis were considered for the individual and community level model adjustments.

### Model building

A total of four models were fitted. The first was the null model with no exposure variables that were used to verify the variation in the community and give evidence to evaluate random effects at the level of community. The second model was the adjustment of the multiple variable models for individual variables and the third model was adjusted to consider factors at the community level. Whereas, in the fourth model, potential candidate variables from individual and community variables were adjusted to the outcome variable.

### Parameter estimation method

Fixed effects were used to estimate the association between the probability of good vitamin A consumption explanatory variables at community and individual levels and have been expressed as an odds ratio with a 95% confidence interval. For measures of variation (random effects), the intracluster correlation coefficient (CCI), the proportional variation of community variance (VCP), and the median odds ratio (MOR) were used. The MOR is defined as the median of the odds ratio between the zone of greatest risk and the zone with the lowest risk when two zones are randomly selected. The purpose of the Median Odds Ratio (MOR) is to translate the area level variance in the widely used odds ratio (OR) scale that has a consistent and intuitive interpretation.

It is computed by; MOR = exp[√(2×Va)×0.6745] [32]

Where; VA is the area level variance, and 0.6745 is the 75th centile of the cumulative distribution function of the normal distribution with mean 0 and variance 1. See elsewhere for a more detailed explanation (24). Whereas the proportional change in variance is calculated as [33]

PCV=[(VA‐VB)/VA]*100;

Where; where VA = variance of the initial model, and VB = variance of the model with more terms.

## Result

**Fig 1:**
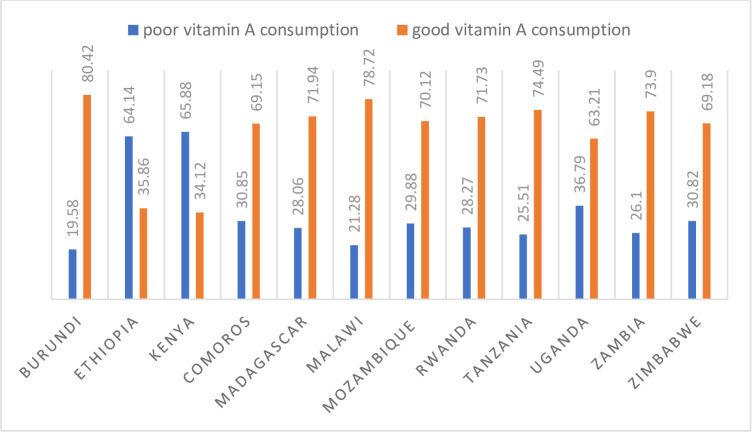
The proportion of vitamin consumption in the 12 East Africa Countries from a recent Demographic and Health survey from 2008 to 2017.

**Fig 2:**
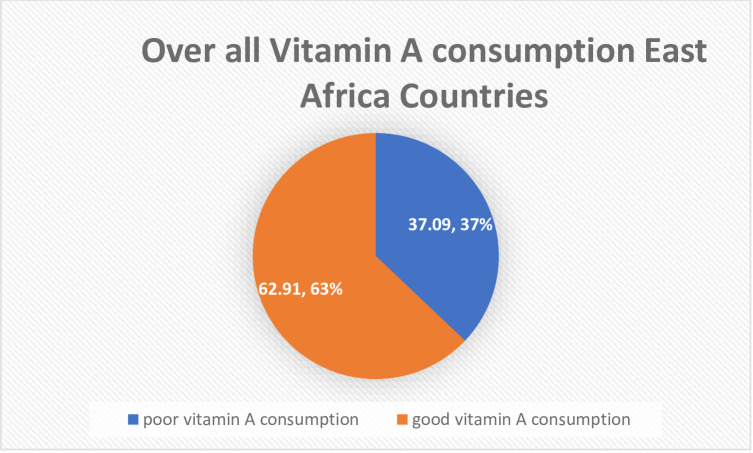
The overall proportion of vitamin A consumption in East Africa Countries from a recent Demographic and Health survey from 2008 to 2017.

A total of 32,275 children aged 6 to 23 months in the last 10 years of the East Africa Countries were included in this study. Of these 20,304(62.91%) with a 95% confidence interval, 62.37% to 63.43% consumed good vitamin A in the 12 East Africa Countries. The largest number of children age 6 to 23 months 5,576 (17.28%) were from Kenya and the smallest number of children aged 6 to 23 months 870 (2.70%) were from Comoros. The majority of the 24,765 (76.73%) respondents were rural residents. The median age group of study respondents was 28 years with an interquartile range of 24 to 34 years with almost half of them 14,976 (46.40%) under the category of the age group of 25 to 34. The majority of study participants 23,436 (72.61%) were married. Almost half of the study participants 16,162 (50.08%) had a primary level of education. The higher proportion of good vitamin A consumption 80.84% was recorded in Burundi and the smallest good vitamin A consumption recorded at 34.12% was recorded in Kenya (**Table** 1**, Figs 1 and 2**).

**Table 1:** Individual and Country level distribution of variables in 12 East Africa Countries from 2008 to 2017.

Variables	Frequency(N = 32,275)	Percent
**Vitamin A consumption(outcome)**		
Good vitamin A consumption	20,304	62.91
Poor vitamin A consumption	11,970	37.09
**Country**		
Burundi	4,323	13.40
Ethiopia	3,240	10.04
Kenya	5,576	17.28
Comoros	870	2.70
Madagascar	1,725	5.35
Malawi	1,641	5.09
Mozambique	3,500	10.85
Rwanda	1,115	3.71
Tanzania	3,085	9.55
Uganda	1,429	4.43
Zambia	3,811	11.81
Zimbabwe	1,877	5.82
**Residence**		
Urban	7,510	23.27
Rural	24,765	76.73
**Age group**		
15–24	11,300	30.83
25–34	14,976	46.40
35–49	23,132	18.59
**Marital status**		
Single	8,339	27.39
Married	23,436	72.61
**Educational status**		
No education	8,275	25.08
Primary	16,162	50.08
Secondary	6,711	20.80
Higher	1,124	3.48
**Literacy level**		
Cannot read	12,288	38.11
Can read	19,957	61.89
**Maternal Occupation(N = 29,377)**		
Had no occupation	9,613	32.72
Had Occupation	19,764	67.28
**Wealth index**		
Poor	14,784	45.81
Middle	6,385	19.79
Rich	11,104	34.41
**Child age in month**		
6–8	5,554	17.21
9–11	5,455	16.90
12–17	11,250	34.84
18–23	10,014	31.03
**Media exposure**		
No	22,396	69.39
Yes	9,879	30.61
**Family size**		
< = 3	4,911	15.22
>3	27,364	84.78
**Breastfeed**		
Yes	24,297	75.29
No	7,975	24.71
**Parity**		
1	7,087	21.96
2–4	15,773	48.87
5+	9,414	29.17

### Multilevel logistic regression analysis

#### The random-effects analysis result

Fixed effects and random interceptions for good vitamin A intake are shown in Table 1. The results of the null model (Model I) showed statistically significant variability in the odds of vitamin A intake with community variance (t = 0.417, p-value = p < 0.001). Likewise, the ICC in the null model implied that 11.27% of the total variance of good vitamin A intake was due to differences between communities. While community variance was expressed as the intra-cluster correlation coefficient (ICC) and the median odds ratio (MOR). The MOR was also 2.11 (95% CI 1.98.2.26). This suggests that the likelihood of having a child with good vitamin A intake was 2.11 times higher when respondents moved from low-risk to high-risk communities. This has shown a significant heterogeneity in vitamin A consumption between different communities. In the full model (adjusted for individual and community factors), the community variance (community variance = 0.082; SE = 0.012; P-value, 0.001) remained significant but decreased. Approximately 2.45% of the total variance of good vitamin A intake that can be attributed to contextual factors remained significant even after accounting for some contextual risk factors. The proportional change in variance (PCV) in this model was 80.33 percent, indicating that 80.33 percent of the community variance observed in the null model was explained by the community as well as individual variables.

#### The fixed effects analysis result

The model with smaller deviance (model IV) best fits the data and the interpretation of the fixed effects was based on this model. Model four was adjusted for both individual and community-level factors. Consequently, respondents’ age group, marital status, educational status, literacy level, respondent occupation, wealth index, media exposure, children’s age in the month, parity, and living Country have been significantly associated with good vitamin A consumption in East Africa Countries.

The odds of good vitamin A consumption among women aged 25–34 and 35–49 were increased by 22% and 41% as compared to the age group 15–24 (AOR = 1.22;95%CI:1.13,1.32), and (AOR = 1.41;95%CI:1.27,1.57) respectively. The odds of good vitamin A consumption increase by 20% among married as compared to a single (AOR = 1.20; 95%CI: 1.12, 1.28). Education has a significant effect on the consumption of vitamin A consumption. The odds of good vitamin A consumption among secondary education level mothers increase by 15% as compared to non-educated mothers (AOR = 1.15; 95%CI: 1.03, 1.28). The odds of good vitamin A consumption among literate mothers increased by 18% as compared to illiterate (AOR = 1.18; 95%CI: 1.10, 1.27). The odds of good vitamin A consumption among mothers who had occupation increased by 41% as compared to mothers with no occupation (AOR = 1.41; 95%CI: 1.33, 1.50). The odds of good vitamin A consumption among women in the middle and rich wealth category increase by 23% and 38% as compared to women with poor wealth status (AOR = 1.23;95%CI:1.14,1.33) and (AOR = 1.38;95%CI:1.26,1.47) respectively. Children’s age in moth had a significant effect on the good consumption of vitamin A. The odds of good vitamin A consumption among children of age in months 9–11,12–17, and 18–23 were 2.91,4.46 and 4.83 times higher as compared to children of age in months 6–8 (AOR = 2.91;95%CI:2.67,3.18), (AOR = 4.46;95%CI:4.13,4.82), and (AOR = 4.83;95%CI:4.45,526) respectively. The odds of good vitamin A consumption among media-exposed mothers increase by 18% as compared to media-non-exposed mothers (AOR = 1.18;95%CI:1.11,1.26). There was a relationship between parity and good vitamin A consumption. The odds of good vitamin A consumption among parity of 2–4 and 5+ were decrease by 23% and 31% as compared to parity 1 (AOR = 0.77;95%CI:0.70,0.83), and (AOR = 0.69;95%CI:0.62,0.78) respectively. Living countries have a significant effect on good vitamin A consumption in East Africa Countries. Burundi was taken as a reference since there is better performance on good vitamin A consumption as compared to other East Africa Countries. The odds of good vitamin A consumption among children of age 6 to 23 months in Ethiopia decreased by 88% as compared to children live Burundi (AOR = 0.12;95%CI:0.10,0.13) (Table 2).

**Table 2:** Multivariable multilevel logistic regression analysis of both individual and community-level factors associated with vitamin A consumption in East Africa Countries from 2008 to 2017.

Individual and community-level variables	Models			
	Null model	Model I	Model II	Model III
	AOR (95%CI)	AOR (95%CI)	AOR (95%CI)	AOR (95%CI)
**Age group**				
15–24		1		1
25–34		1.19(1.10,1.28)		1.22(1.13,1.32)*
35–49		1.40(1.26,1.55)		1.41(1.27,1.57)*
**Marital status**				
Single		1		1
Married		0.99(0.94,1.06)		1.20(1.12,1.28)*
**Educational status**				
No education		1		1
Primary		1.30(1.21,1.40)		1.08(0.99,1.28)
Secondary		1.37(1.24,1.51)		1.15(1.03,1.28)*
Higher		1.04(0.87,1.24)		1.19(0.98,1.43)
**Literacy level**				
Cannot read		1		1
Can read		1.28(1.19,1.37)		1.18(1.10,1.27)*
**Maternal Occupation(N = 29,377)**				
Had no occupation		1		1
Had Occupation		1.84(1.74,1.95)		1.41(1.33,1.50)*
**Wealth index**				
Poor		1		1
Middle		1.29(1.19,1.38)		1.23(1.14,1.33)*
Rich		1.37(1.29,4.95)		1.38(1.26,1.47)*
**Child age in month**				
6–8		1		1
9–11		2.80(2.58,3.05)		2.91(2.67,3.18)*
12–17		4.06(3.77,4.38)		4.46(4.13,4.82)*
18–23		4.56(4.21,4.95)		4.83(4.45,5.26)*
**Media exposure**				
No		1		1
Yes		1.16(1.09)		1.18(1.11,1.26)*
**Family size**				
< = 3		1		1
>3		1.05(0.96,1.24)		1.05(0.96,1.14)
**Breastfeed**				
No		1		1
Yes		1.03(0.95,1.11)		0.96(0.89,1.05)
**Parity**				
1		1		1
2–4		0.82(0.75,0.89)		0.77(0.70,0.83)*
5+		0.74(0.60,0.83)		0.69(0.62,0.78)*
**Residence**				
Urban			1	1
Rural			0.79(0.75,0.84)	0.96(0.89,1.04)
**Country**				
Burundi			1	1
Ethiopia			0.11(0.10,0.12)	0.12(0.10,0.13)*
Kenya			0.09(0.08,0.10)	0.36(0.32,0.41)*
Comoros			0.48(0.40,0.57)	0.56(0.47,0.68)*
Madagascar			0.64(0.56,0.73)	0.67(0.58,0.77)*
Malawi			0.88(0.76,1.02)	0.91(0.78,1.06)
Mozambique			0.47(0.42,0.53)	0.57(0.51,0.65)*
Rwanda			0.60(0.51,0.69)	0.53(0.45,0.63)*
Tanzania			0.71(0.63,0.79)	0.66(0.59,0.75)*
Uganda			0.40(0.35,0.45)	0.41(0.35,0.47)*
Zambia			0.60(0.54,0.67)	0.66(0.58,0.75)*
Zimbabwe			0.44(0.39,0.50)	0.41(0.35,0.47)*
**Random effects**				
Community variance (SE)	0.417(0.034)	0.090(0.012)	0.078(0.011)	0.082(0.012)*
ICC%	11.27%	2.66%	2.32%	2.45%
PCV%	1	78.41%	81.29%	80.33%
MORE	2.11(1.98,2.26)	1.41(1.38,1.45)	1.38(1.18,1.61)	1.39(1.36,1.42)*
**Model comparison**				
LLR	-21,091	-16600	-18,751	-15,772
Deviance	42,182	33,200	37,502	31,544

AOR: Adjusted odds ratio; 1: Refference category MOR: Median odds ratio PCV: Proportional change in variance; ICC: Interclass Correlation * = significant at 5%; MOR: Median Odds Ratio.

## Discussion

The main objective of this study was to know the magnitude and determinants of good vitamin A consumption in East African countries. Both bivariable and multilevel logistic regression model was fitted. From the multilevel logistic regression model women’s age, marital status, maternal education, wealth index, maternal occupation, children’s age in a month, media exposure, literacy rate, and parity were significantly associated with good vitamin A-rich foods consumption in East Africa.

The magnitude of good vitamin A consumption was 62.91% with a 95% CI of 62.37 to 63.43. This finding is higher than from a study done at Mali DHS and Ethiopia [1,17,28]. The difference in results can be explained by the effect of sample size, differences in the study population, tools used to measure the outcome variables across the studies, and study settings differences.

In this study, good vitamin A consumption among women aged 25–34 and 35–49 was higher than among the age group 15–24. This might be due to the higher age group might give them more chances to receive a lot of pertinent information about vitamin A and that might lead them to consume better than their counterparts. Furthermore, the higher age group is more likely to be employed while studies showed that being employed was found to increase the chance of good vitamin A consumption as than non-employed ones [34,35]. This might be explained as the information needed to consume vitamin A adequately can be easily obtained from their colleagues; In addition, the employed women were mostly educated, and it improves the use of health information like advantages and sources of vitamin A-rich foods.

Good vitamin A consumption is higher among married respondents as compared to single ones. This might be attributed to the additional chance of getting support from their husband and if the husband is educated, better consumption of vitamin A-rich foods is likely to occur in that household whereas studies showed that the presence of an educated husband in the household was associated with better consumption of vitamin A [35–37]. Moreover, living with a husband may give a chance to earn a better income and strengthen the financial capability of that family and that will in turn improves the capability of consuming vitamin A-rich foods, which are expensive to be consumed for people with low income [23].

This study indicates that good vitamin A consumption was higher among literate mothers including primary education level mothers increase as compared to illiterate mothers. Intakes of preformed vitamin A from animal and fortified sources as well as vitamin A density were strongly linked to education, with a higher intake among those with a higher level of education [38]. This finding is also supported by different studies where education improves the use of vitamin A supplementation [11,13,28,35–37]. Thus, mothers who are educated are more likely to get more information about vitamin A food sources than illiterate ones.

Furthermore, good vitamin A consumption was higher among mothers who had occupations than mothers with no occupation and this is consistent with a study finding [34]. This might be due to the information needed to consume vitamin A-rich foods being easily obtained from their colleagues and mothers who had occupations are more educated than mothers who had no occupation.

This study showed that good vitamin A consumption was higher among women in the middle and rich wealth category compared to women with poor wealth status. This finding is supported by studies; [11,13,17,19,28] and this might be due to the higher chance of consumption of Vitamin A rich foods among those higher wealth status than poor wealth status as they may not be afford to buy vitamin A-rich foods which are expensive for people with low income [23].

Children aged greater than 9 months had a significant effect on the good consumption of vitamin A than children of age in months 6–8. This is similar to a study finding a study in Bangladesh [36]. This result is probably due to the numerous carrying attitudes of parents regarding what types of foods they usually have to feed their children in different age groups. Another explanation may be also the reduced intake of breast milk with increasing age, and the bad quality of breast milk due to poor nutrition of the mother.

Good vitamin A consumption is higher among media-exposed mothers increase than media non-exposed mothers. This was supported by studies that showed that there was a positive relationship between media exposure and vitamin A consumption [34,37]. This is because the information needed to consume vitamin A-rich foods can be obtained easily through the utilization of media.

This study showed that good vitamin A consumption was lower among parity of greater than two as compared to parity of one. This was similar to a study done in Ethiopia that indicated a significant association between smaller family sizes and good vitamin A-rich foods [28]. The possible explanation for this finding is due to a challenge a mother face to feed vitamin-rich foods to a larger family than a smaller family size due to the expense of vitamin A-rich foods. Whereas feeding vitamin-rich foods to large family sizes is more challenging due to the inflation of the cost of living.

Good vitamin A consumption among children of age 6 to 23 months in Ethiopia decreased by 88% as compared to children live Burundi. The difference might be attributed due to the difference in socioeconomic and study settings.

## Conclusion

The magnitude of good vitamin A consumption in twelve East African countries is low. The respondents with an age group of 25–34 and 35–49, married, literate, had occupation, middle and higher wealth index, have media exposure, children aged nine-month to 2 years, 2–4 and 5+ parity and different living Country was significantly associated with good vitamin A consumption in the East Africa Counties. Therefore, policymakers should give due attention to the age group 15–24, single, illiterate, jobless, poor wealth status, children of age in months 6–8, media non-exposed mothers, and parity one to improve good consumption of foods rich in vitamin A in East Africa countries.

## Supporting information

S1 Dataset (XLS)
